# Association of *NQO1* levels and its genetic polymorphism with susceptibility to methamphetamine dependence

**DOI:** 10.1002/mgg3.1789

**Published:** 2021-09-01

**Authors:** Huan Liu, Wei Zhang, Xiao‐Dong Deng, Ying Ma, Yun Liu

**Affiliations:** ^1^ Department of Preventive Medicine North Sichuan Medical College Nanchong Sichuan China; ^2^ Department of Forensic Medicine North Sichuan Medical College Nanchong Sichuan China; ^3^ Department of Neurology Affiliated Hospital of North Sichuan Medical College Nanchong Sichuan China; ^4^ Sichuan Key Laboratory of Medical Imaging North Sichuan Medical College Nanchong Sichuan China

**Keywords:** genetic susceptibility, methamphetamine dependence, *NQO1*, SINGLE‐nucleotide polymorphism

## Abstract

**Background:**

The quinone oxidoreductase 1 (*NQO1*) gene was involved in the pathophysiological process of illicit drugs abuse, and its polymorphisms might be associated with methamphetamine (METH) dependence susceptibility. The purpose of this study was to examine the *NQO1* mRNA and protein levels and to analyze the 609C/T polymorphism (rs1800566) between METH‐dependent patients and controls.

**Methods:**

A total of 392 METH‐dependent patients (cases) and 669 healthy controls (controls) were enrolled in the study. The quantitative real‐time polymerase chain reaction (RT‐qPCR) and enzyme‐linked immunosorbent assay (ELISA) were used to detect the relative expressions of *NQO1* mRNA in PBMCs and protein levels in plasma, respectively. PCR‐restriction fragment length polymorphism (RFLP‐PCR) and direct‐sequencing genotyping were used to detect the alleles and genotypes of *NQO1* 609C/T polymorphism.

**Results:**

The levels of *NQO1* mRNA in cases (3.2650 ± 2.2943) was significantly higher than in controls (1.0125 ± 0.7959) (*p* < 0.001), the plasma protein in cases (0.2368 ± 0.1486) was significantly lower than in controls (0.5844 ± 0.1742) (*p* < 0.001). The T allele of the 609C/T polymorphism significantly increased the risk of METH dependence (*p* = 0.032, OR = 1.214, 95%CI = 1.017–1.450). The TC and TC/TT genotypes of 609C/T were observed significantly more frequently in cases than in controls, respectively (TC vs CC: *p* = 0.012, OR = 1.457, 95% CI = 1.087–1.952; TC/TT vs CC: *p* = 0.008, OR = 1.460, 95% CI = 1.102–1.935). Similar results were obtained after adjusting for age and sex. We failed to find that any genotype of 609C/T polymorphism affected the mRNA or plasma protein levels in controls, respectively (*p* > 0.05).

**Conclusion:**

The findings suggested that *NQO1* might play an important role in the pathophysiological process of METH dependence, and the 609C/T polymorphism might contribute to the susceptibility to METH dependence in a Chinese Han population.

## Graphical abstract

Our results indicated that *NQO1* mRNA levels increased in PBMCs and its plasma protein levels decreased in METH dependence. And, the T allele and carried T genotypes of *NQO1* 609C/T polymorphism increased the risk of METH dependence.

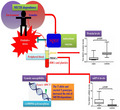

**Highlights**

1. The NQO1 may play an important role in the pathophysiological process of METH dependence.
2. The *NQO1* rs1800566 polymorphism (609C/T) significantly increased METH dependence risk.
3. Individuals with any of the genotypes of rs1800566 did not affect its mRNA and protein levels, respectively.

## 1 INTRODUCTION

Methamphetamine (METH) was one of the most abused drugs in China, and long‐term abusing of it may result in weakened immunity and susceptibility to infection, which would affect the activities of nuclei of the central nervous system and higher functions of the nervous system, such as rewarding, learning, and memory (Friedman et al., 2003). METH dependence was considered to be a chronic recurrent brain dysfunction disease involving many genetic and environmental factors. Genetic factors were topics of major interest that contributed to the etiology of substance abuse and dependence (Crabbe, 2002; Kendler, 2001; Tsuang et al., 2001; Uhl et al., 2002). The METH dependence association with susceptibility genes has not been fully elucidated. The currently known susceptibility genes only can explain a small fraction in the inherited risk of METH dependence. Oxidative stress plays an important role in the mechanisms of METH dependence neurotoxicity. METH‐intake could cause dopamine release and then produce dopamine‐quinones and additional reactive oxygen species by auto‐oxidizing (Ohgake et al., 2005). Quinone oxidoreductase 1 (*NQO1*+125860) also known as diphtheria toxin diaphorase (DT‐diaphorase) is located on chromosome 16q22.1. It is widely distributed in the most eukaryotic cell cytoplasm, such as epithelial cells, endothelial cells, various tumor cells, nerve cells, and glial cells, etc. (Fryatt et al., 2004; Schlager & Powis, 1990; Siegel & Ross, 2000). *NQO1*, considered as an antioxidant enzyme, is the main factor for antioxidant damage and cells protection by catalyzing a two electron reduction of quinone compounds and detoxification of the electrophilic compounds (Long & Jaiswal, 2000; Ross et al., 2000; Sorensen et al., 2003). So the gene coding for *NQO1* was therefore being investigated in relation to the progression of METH dependence. *NQO1* was likely to be a positional candidate gene of illicit drug dependence.

Single‐nucleotide polymorphisms (SNPs) are the most frequent type of variation and powerful tools for medical genetic studies (Wang et al., 1998). The *NQO1* gene genetic variations or polymorphisms that produce a functionally altered enzyme may also affect the risk of different diseases, including METH dependence (Hubackova et al., 2009; Ohgake et al., 2005; Tijhuis et al., 2008). To date, associations between *NQO1* SNPs and multiple kinds of diseases have been widely studied, especially the rs1800566 (609C/T), a cytosine (C) to thymine (T) change at nucleotide position 609 in exon 6 which resulted in a proline‐to‐serine amino acid change at codon 187 of the amino acid sequence of the protein. In comparative studies with wild‐type protein, the purified mutant *NQO1* had only 2% of wild‐type activity (Ross et al., 1996; Traver et al., 1997). To our knowledge, the correlation between *NQO1*, the rs1800566 polymorphism, and METH dependence was rarely reported. Therefore, we hypothesized that *NQO1* may play an important role in the pathophysiological process of METH dependence. We performed a study to investigate whether *NQO1* mRNA and protein levels and the 609C/T polymorphism of *NQO1* were associated with susceptibility to METH dependence in a general Chinese Han population.

## 2 MATERIALS AND METHODS

### 2.1 Study samples

This study was approved by the Human Ethics and Research Ethics committees of North Sichuan Medical College, and all participants or their next of kin provided written informed consent to participate in this research. All participants were unrelated Chinese Han ethnic origin people by self‐description.

This was a case–control study including 392 METH‐dependent patients (cases) and 669 healthy individuals (controls), who were recruited from the Drug Rehabilitation Center of Nanchong and the Affiliated Hospital of North‐Sichuan Medical College from March 2017 to October 2018, respectively. In brief, diagnostic criteria of METH dependence was confirmed according to ICD‐10 criteria (F15.201) and the Diagnostic and Statistical Manual of Mental Disorders, Fifth Edition (DSM‐5) (American Psychiatric Association, 2013) for amphetamine‐type stimulants (ATS) dependence, who were patients without polysubstance abuse of drug rehabilitation center of Nanchong. We excluded patients with tumors, serious cardiovascular diseases, liver and kidney function failure, sexually transmitted diseases, neurodegenerative diseases, and patients without informed consent. The exclusion criterion of controls was the same as those used in cases.

Other covariates of METH dependence and normal controls were also carefully collected, including demographic information and laboratory auxiliary inspection results.

### 2.2 Quantitative real‐time polymerase chain reaction

Peripheral blood mononuclear cells (PBMCs) were extracted using Ficoll‐Hypaque density gradient centrifugation following the manufacturer's instructions (TBD). Total RNA of the PBMCs were extracted using Trizol reagent (Invitrogen), and using a PrimeScript™ RT Reagent Kit with gDNA Eraser (Takara Bio Inc.) to synthetize cDNA according to the manufacturer's instructions. The SYBR^®^ Premix Ex Taq™ II kit (Takara Bio Inc.) was used in the quantitative real‐time polymerase chain reaction (RT‐qPCR). RT‐qPCR instrument (ABI 7500, Applied Biosystems, Foster City, CA, USA) was used for detection. The relative mRNA expression levels of *NQO1* were tested by the comparative *Ct* method using 2^−ΔΔ^ *^Ct^* (Δ*Ct* = *Ct^NQO1^*−*Ct* ^β−actin^; ΔΔ*Ct* = Δ*Ct* ^cases^−ΔCt^controls^). *NQO1* gene (NG_011504.2) primers were as follows: sense primer: 5′‐AAT GAA GGG ATT GGA CCG AGC‐3′ and anti‐sense primer: 5′‐CAC CCA GCC GTC AGC TAT TGT‐3′ (size: 103 bp). The internal control, β‐actin, were as follows: sense primer: 5′‐CCA CGA AAC TAC CTT CAA CTC C‐3′ and anti‐sense primer: 5′‐GTG ATC TCC TTC TGC ATC CTG T‐3′ (size: 132 bp). PCR amplification was performed in a volume of 20 μl containing 6.8 μl ddH_2_O, 0.4 μl ROX Reference Dye, 10 μl SYBR Premix Ex Taq (×2), 0.4 μl forward primer and reverse primer, respectively, and 2 μl cDNA. The amplification cycling parameters were 95℃ for 30 s by one cycle, 95℃ for 5 s, 60℃ for 30 s by 40 cycles, followed melting reaction at 95℃ for 10 s, 65℃ for 60 s, 97℃ for 1 s by one cycle. All tests were in triplicate and repeated at least three times, samples without a template served as a blank control. The standard curve was produced by serial dilution of a plasmid containing *NQO1* or β‐actin cDNA. All of the above operations were carried out with reference to document of Ma et al. (2016). In the study, we have chosen 84 METH dependence and 84 controls randomly for analyzing *NQO1* mRNA expression.

### 2.3 Enzyme‐linked immunosorbent assay

The plasma was stored at −80°C until analysis that gradiently thawed to room temperature. The *NQO1* plasma levels were detected by using commercial enzyme‐linked immunosorbent assay (ELISA) kits according to the manufacturer's instruction (USCN). The detectable sensitivity of *NQO1* ELISA kit was 0.124 ng/ml and the detection range was 0.312–20 ng/ml. The Benchmark microplate reader was used to measure the absorbance at 450 nm (Bio‐Rad). The mean absorbance in the plasma samples of the negative controls was used as a cutoff value. Concentrations were calculated by standard curves. All tests were in triplicate and repeated at least three times. The ELISA samples were the same as those used for the qPCR.

### 2.4 *NQO1* 609C/T genotyping

**FIGURE 1:**
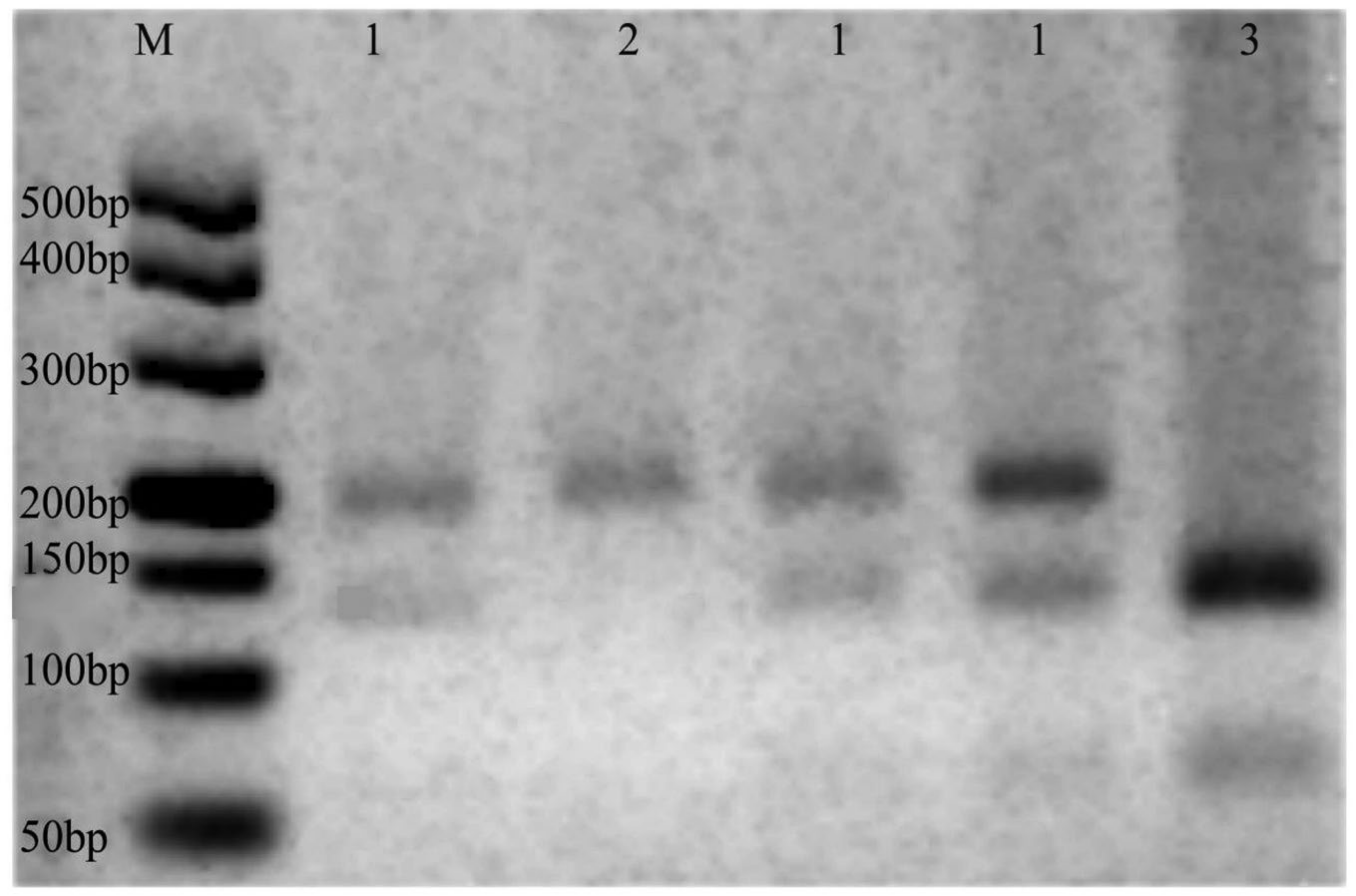
Restriction fragment size of the NQO1 609C/T locus polymorphism. M: DNA markerⅠ; 1: TC genotype; 2: CC genotype; 3: TT genotype. NQO1 gene (NG_011504.2)

Genomic DNA was extracted from blood samples by a commercial DNA isolation kit according to the manufacturer's instructions (BioTeke). The polymorphism of a C to T substitution in exon 6 of the *NQO1* gene (rs1800566) was identified by PCR restriction fragment length. PCR primers were as follows: forward primer, 5′‐AAG CCC AGA CCA ACT TCT‐3′ and reverse primer, 5′‐ATT TGA ATT CGG GCG TCT GCT G‐3′ (size: 174 bp). The total volume of PCR was 25 μl, containing 12.5 μl of 2 × Taq PCR MasterMix (Tiangen Biotech), 9.9 μl of ddH_2_O, 2 μl of DNA, and 0.3 μl of each primer. The PCR conditions were 94℃ for 5 min, followed by 40 cycles of 30 s at 94℃, 30 s at 66℃, and 30 s at 72℃, with a final elongation at 72℃ for 5 min and 4℃ for keeping warm, in a MyCycler™ Thermal Cycler (Bio‐Rad, USA). 0.3 μl HinfI (New England BioLabs Ltd) was used to digeste the PCR products at 37℃ for 7 h in a reaction volume of 10 μl. The PCR products were cut by the restriction enzyme of HinfI, producing two fragments of 119 bp and 55 bp for the T/T genotype, three fragments of 174 bp, 119 bp, and 55 bp for the C/T genotype, and one fragment of 174 bp for the C/C genotype (Figure 1). To confirm the accuracy of the method used, the genotypes were confirmed by DNA sequencing analysis (ABI3730xl, USA). Repeat assays were performed on about 10% of the samples which were randomly selected, and the results were 100% concordant.

### 2.5 Statistical analyses

SPSS version 13.0 was used for statistical analyses and a two‐sided *p* value lower than 0.05 was considered as statistical significance. The mean ± standard deviation (SD) was used to express continuous variables, and numbers or percentage (%) was used to express categorical variables. The *χ* ^2^ test or Fisher's exact test for categorical variables or Student's *t*‐test or rank sum test for continuous variables was used to evaluate the differences between the cases and controls. The odds ratio (OR) and 95% confidence intervals (CI) were used to estimate associations between the polymorphism and METH dependence through both univariate and multivariate logistic regression analyses. The *χ* ^2^ test or Fisher's exact test was used to estimate stratified analyses in cases. The mRNA and protein levels were compared between genotypes or alleles of the *NQO1* 609C/T polymorphism by Student's *t*‐test in controls, respectively. The *χ* ^2^ test was used to estimate Hardy–Weinberg equilibrium (HWE) in controls.

## 3 RESULTS

### 3.1 Characteristics of the study population

The demographics and laboratory tests of the study population are summarized in Table 1. The mean age (±SD) of METH dependence (326 males and 66 females) was 29.16 (±7.91) years old, and the control group (529 males and 140 females) was 30.09 (±8.48) years old. There were no differences regarding age (*p* = 0.069) and gender (*p* = 0.104) between METH dependence and controls, respectively. The BMI of METH dependence (20.44 ± 5.14 kg/m^2^) was significantly smaller than controls (22.19 ± 3.13kg/m^2^) (*p* < 0.01). Significant differences were found between the METH dependence and controls in terms of white blood cell, hemoglobin, and diastolic pressure (*p* < 0.01).

**TABLE 1:** Comparison of demographic characteristics and laboratory tests of METH‐dependent group and control group

Variables	METH dependents (n = 392)	Controls (n = 669)	*p* values
Demographics			
Age (Mean ± SD)	29.16 ± 7.91	30.09 ± 8.48	0.069
Male sex, n (%)	326(83.16)	529(79.07)	0.104
Laboratory tests			
BMI (Mean ± SD)	20.44 ± 5.14	22.19 ± 3.13	<0.001
WBC (Mean ± SD)	9.33 ± 2.56	6.01 ± 1.50	<0.001
RBC (Mean ± SD)	5.03 ± 0.60	4.96 ± 0.52	0.099
HGB (Mean ± SD)	152.41 ± 17.34	148.48 ± 17.03	0.002
Systolic pressure (Mean ± SD)	120.20 ± 14.14	120.15 ± 12.07	0.956
Diastolic pressure (Mean ± SD)	77.08 ± 10.76	74.36 ± 9.87	<0.001

### 3.2 Association between *NQO1* mRNA and protein levels and METH dependence

**FIGURE 2:**
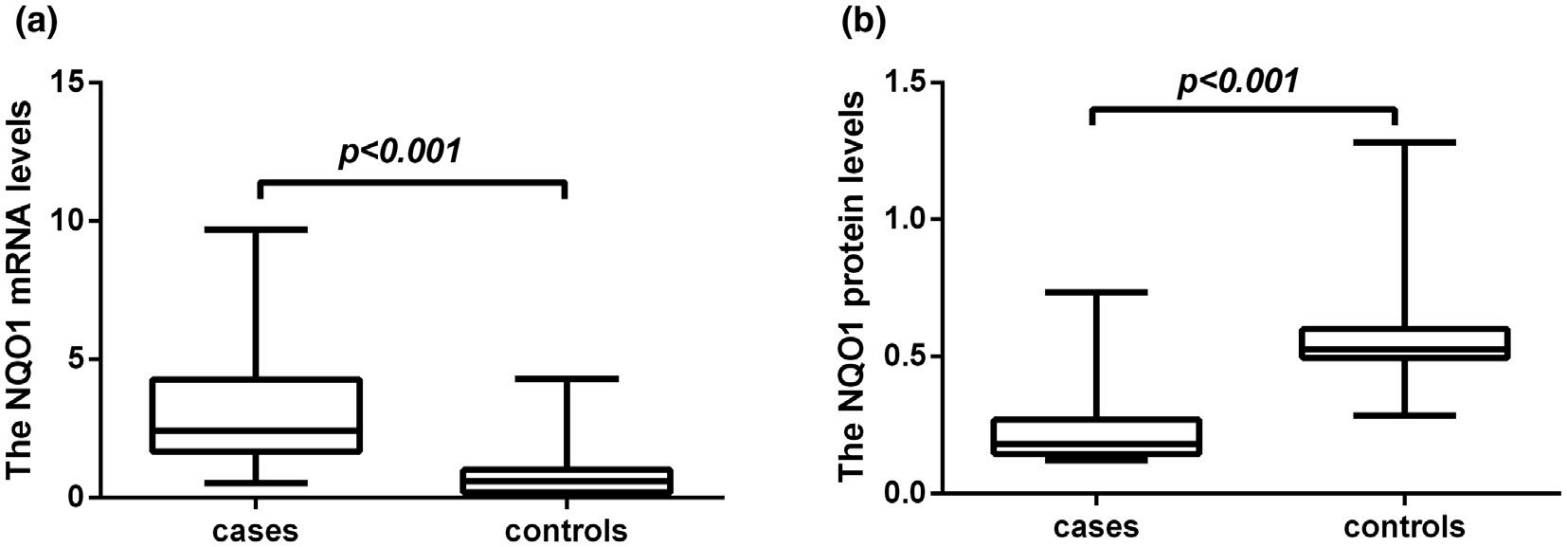
The mRNA and plasma protein levels of quinone oxidoreductase 1 (*NQO1*) in methamphetamine (METH)‐dependent patients (cases) and controls. (a) The *NQO1* mRNA relative expression levels of METH dependence were significantly higher than controls in peripheral blood mononuclear cells (PBMCs) (*p* < 0.001). (b) The *NQO1* plasma protein levels in METH dependence were significantly lower compared with those in controls (*p* < 0.001)

The *NQO1* mRNA expression levels in PBMCs were 3.2650 ± 2.2943 and 1.0125 ± 0.7959 between METH‐dependent patients and controls, respectively. The *NQO1* mRNA levels in METH‐dependent patients were significantly greater than controls (*p* < 0.001, Figure 2a).

The *NQO1* plasma protein levels were 0.2368 ± 0.1486 and 0.5844 ± 0.1742 between METH‐dependent patients and controls, respectively. The *NQO1* plasma protein levels in METH‐dependent patients were significantly lower than controls (*p* < 0.001, Figure 2b).

### 3.3 Association of the *NQO1* 609C/T Polymorphism with METH dependence

The *NQO1* 609C/T polymorphism was successfully genotyped in 392 METH‐dependent patients and 669 controls. The *NQO1* 609C/T polymorphism genotype distribution in controls was in agreement with that expected under HWE (*p* = 0.06). It was indicated that our samples can represent the area. The frequencies of genotypes and alleles in METH dependence and controls are shown in Table 2. The C and T allele frequencies of *NQO1* 609C/T were 53.44% and 46.56% in METH dependence, 58.22% and 41.78% in controls, respectively. The T allele frequencies of *NQO1* 609C/T polymorphism were higher in the METH dependence than controls, which indicated T allele increased the METH dependence risk (*p* = 0.032, OR = 1.214, 95%CI = 1.017–1.450). We performed to assess the strength of association between genotypes of 609C/T polymorphism and METH dependence risk under homogeneous (TT vs CC), heterogeneous (TC vs CC), dominant (TC+TT vs CC), and recessive (TT vs TC+CC) genetic model. Significant differences were found between the METH dependence and controls in heterogeneous (TC vs CC), dominant (TC +TT vs CC) genetic model (TC vs CC: *p* = 0.012, OR = 1.457, 95%CI = 1.087–1.952; TC+TT vs CC: *p* = 0.008, OR = 1.460, 95%CI = 1.102–1.935). There were no significant differences between the METH dependence and controls in homogeneous (TT vs CC), recessive (TT vs TC+CC) genetic model (TT vs CC: *p* = 0.051, OR = 1.472, 95%CI = 0.999–2.168; TT vs TC+CC: *p* = 0.418, OR = 1.147, 95%CI = 0.822–1.601). Similar results were also obtained after adjusting for age and sex by logistic regression analysis.

**TABLE 2:** The allele and genotype frequency of NQO1 609C/T between experimental group and control group

	Allele or Genotype	Case n = 392 (%)	Control n = 669(%)	OR(95%CI)	*p*	Adjust OR^a^ (95%CI)	Adjust OR *p*
	Allele						
	C	419 (53.44)	779 (58.22)	1.00(Ref)			
	T	365 (46.56)	559 (41.78)	1.214 (1.017–1.450)	0.032		
Genetic model	Genotype						
	CC	96 (24.49)	215 (32.14)	1.00(Ref)		1.00(Ref)	
heterogeneous	CT	227 (57.91)	349 (52.17)	1.457(1.087–1.952)	0.012	1.452(1.082–1.948)	0.013
homogeneous	TT	69 (17.60)	105 (15.69)	1.472(0.999–2.168)	0.051	1.423(0.962–2.105)	0.077
dominant	CC	96 (24.49)	215 (32.14)	1.00(Ref)		1.00(Ref)	
	TC+TT	296 (75.51)	454 (67.86)	1.460(1.102–1.935)	0.008	1.445(1.089–1.919)	0.011
recessive	TT	69 (17.60)	105 (15.69)	1.00(Ref)		1.00(Ref)	
	TC+CC	323 (82.40)	564 (84.30)	1.147(0.822–1.601)	0.418	1.110(0.793–1.553)	0.544

Note

*NQO1* gene (NG_011504.2)

Abbreviations: %, frequency of the genotype; CI, confidence interval; *n*, number of subjects; OR, odds ratio; Ref, reference.

^a^ Adjusted for age and gender.

What was more, we also estimated the *NQO1* 609C/T polymorphism associated with demographic characteristics and laboratory tests under heterozygote and homozygote genetic models in METH dependence. We failed to find a significant association between the *NQO1* 609C/T polymorphism and demographic characteristics and laboratory tests (*p* > 0.05) (Table 3).

**TABLE 3:** Hierarchical analysis of NQO1 609C/T polymorphism in experimental group

Variables	TT vs CC		TC vs CC	
	OR (95% CI)	*p* value	OR (95% CI)	*p* value
gende	0.764 (0.305–1.913)	0.564	0.523 (0.258–1.062)	0.07
age	1.342 (0.713–2.523)	0.361	1.169 (0.722–1.892)	0.525
BMI	1.066 (0.505–2.249)	0.867	1.119 (0.593–2.110)	0.729
WBC	0.574 (0.284–1.159)	0.120	0.804 (0.479–1.349)	0.408
RBC	0.779 (0.387–1.565)	0.482	0.832 (0.495–1.398)	0.488
HGB	1.088 (0.540–2.194)	0.814	0.934 (0.557–1.568)	0.797
systolic pressure	0.874 (0.426–1.795)	0.714	1.274 (0.752–2.161)	0.368
diastolic pressure	0.500 (0.237–1.055)	0.067	0.641 (0.377–1.089)	0.099

Note

NQO1 gene (NG_011504.2)

Abbreviations: BMI, body mass index; HGB, hemoglobin; RBC, red blood cell; WBC, white blood cell.

### 3.4 Correlation between 609C/T genotype and *NQO1*

To evaluate the functional relevance of the *NQO1* 609C/T polymorphism, we analyzed the correlation between the *NQO1* mRNA and protein levels and the 609 C/T polymorphism genotypes in 84 healthy individuals by using the aforementioned data and genotyping data.

**FIGURE 3:**
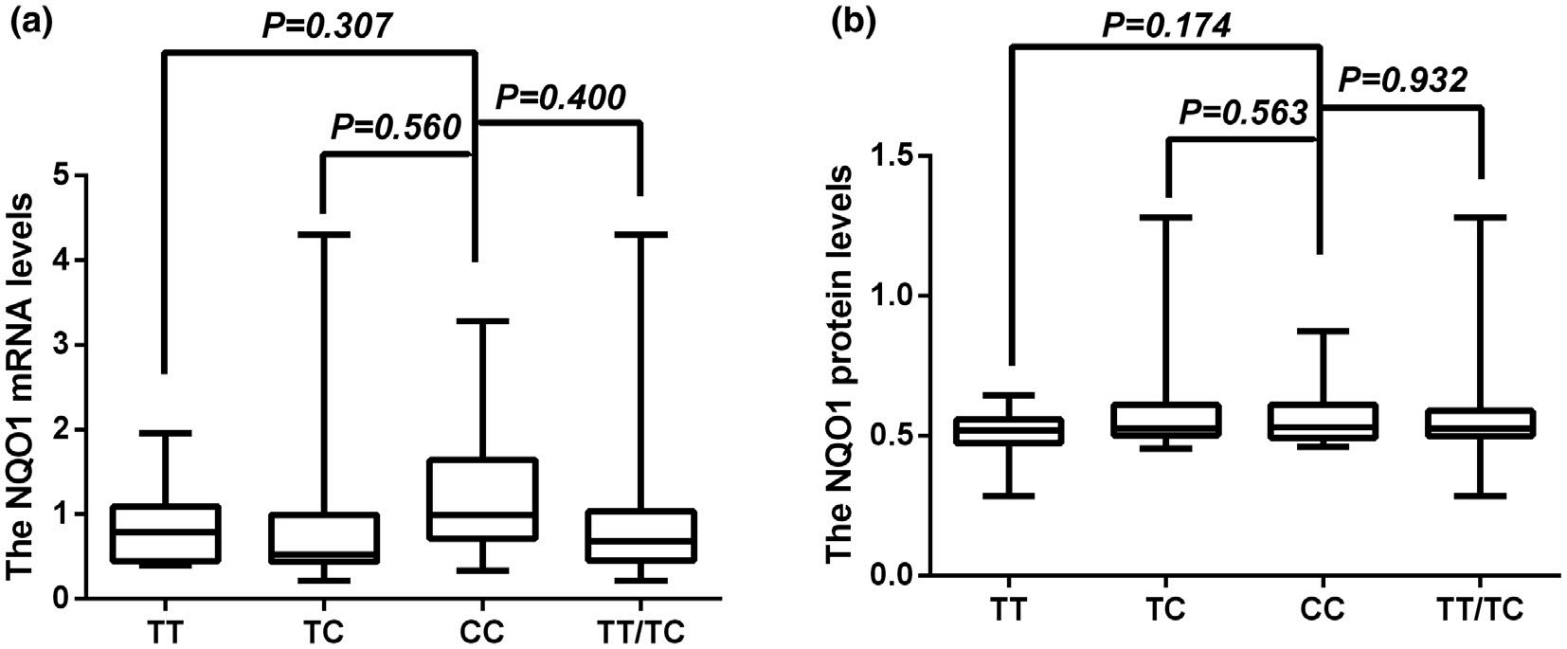
Association of quinone oxidoreductase 1 (*NQO1*) 609C/T polymorphism genotypes and its transcription and translation in controls. (a) None of the genotypes affected mRNA expression levels in peripheral blood mononuclear cells (PBMCs) (*p* > 0.05). (b) None of the genotypes affected plasma protein levels (*p* > 0.05)

We found that the *NQO1* mRNA levels for the TT, TC, CC, and TT/TC genotypes were 0.8751 ± 0.4712, 0.9531 ± 1.0155, 1.1406 ± 0.7389, and 0.9225 ± 0.8336, respectively. Each genotype of the *NQO1* 609 C/T polymorphism did not affect the mRNA levels in PBMCs (TT vs CC: *p* = 0.307; TC vs CC: *p* = 0.560; TT+TC vs CC: *p* = 0.400) (Figure 3a). *NQO1* plasma protein levels for the TT, TC, CC, and TT/TC genotypes were 0.5058 ± 0.1107, 0.6195 ± 0.2257, 0.5814 ± 0.1198, and 0.5863 ± 0.2035, respectively. None of *NQO1* 609C/T genotypes changed plasma protein levels of *NQO1* (TT vs CC: *p* = 0.174; TC vs CC, *p* = 0.563; TT+TC vs CC, *p* = 0.932) (Figure 3b). Similar results were observed in METH dependence (data not shown).

## 4 DISCUSSION

This study suggested that the *NQO1* may play an important role in the pathophysiological process of METH dependence and the T allele of *NQO1* 609C/T polymorphism significantly increased the METH dependence risk. However, the *NQO1* 609C/T polymorphism did not affect the transcription and translation levels of *NQO1*.

In characteristics of the study population, there were no significant differences between the METH dependence and controls in age and gender (*p* > 0.05). It can avoid the influence of age and gender on the results of the experiment and to ensure the comparability of the experimental results between the METH dependence and controls. However, METH‐dependent patients were young with a mean age (±SD) of 29.16 (±7.91) years old, and the males were more than females. This was consistent with other reports (Huang et al., 2011; Liao et al., 2014; Wang et al., 2015). In the current study, significant differences were found in terms of BMI, white blood cell, hemoglobin, and diastolic pressure between the METH dependence and controls, respectively (*p* < 0.05). These indicated that METH dependence may cause changes in body quality and physiological and biochemical indices. The BMI of METH dependence (20.44 ± 5.14kg/m^2^) was significantly lower than controls (22.19 ± 3.13kg/m^2^) (*p* < 0.01). This was consistent with the literature reports (Bluml et al., 2012; Suriyaprom et al., 2011). Studies in the HIV‐infected population also showed that the BMI values of illicit drug abusers were lower than controls (Forrester et al., 2005). There was a significant inverse relationship between obese male addicts and drug dependence in the past years (Barry & Petry, 2009). It was suggested that METH dependence may inhibit food intake or destroy the nutritional balance and affect the absorption of nutrition. Therefore, malnutrition can be caused (Nazrul et al., 2001; Winslow et al., 2007). The white blood cell of METH dependence was significantly higher than controls (*p* < 0.01). It may show that the immunity of METH dependence was decreased and the chance of infection was increased. The hemoglobin of METH dependence was significantly higher than controls (*p* < 0.01), which was similar to the study by Fan et al. (2016) and different from those reported by Liu et al. (2008) who studied the effect of heroin on the hemoglobin, so the difference may be related to the type and manner of drug absorbing. The diastolic blood pressure in the METH dependence was higher than controls (*p* < 0.01), which indicated that long‐term drug abuse may not only lead to the damage of the cardiovascular system but also other tissues and organs, such as poor vascular elasticity, so that it can lead to the increase of diastolic blood pressure.

In the current study, we found that *NQO1* mRNA levels in PBMCs of METH dependence were significantly higher than controls, and plasma protein levels were significantly lower in METH dependence. It indicated that *NQO1* could play an important role in the development, progression, and outcome of METH dependence. Solleti et al. (2017) reported that *NQO1* mRNA expression was higher in nicotine‐vaporized cells than nicotine‐free cells. Yeligar et al. (2010) reported that the expression of *NQO1* mRNA was increased in mouse liver cells treated with alcohol. The increased of *NQO1* mRNA in METH dependence was consistent with the changes induced by nicotine and alcohol. *NQO1* was a multifunctional protein and mainly expressed in the cytoplasm of nerve cells and glial cells, and only a small amount exists in mitochondria, endoplasmic reticulum, and nucleus. It participated in brain oxidative stress by reducing the level of reactive oxygen species in cells and removing a variety of toxic substances (Dinkova‐Kostova & Talalay, 2010; Kensler et al., 2007; Vasiliou et al., 2006; Winski et al., 2002). What was more, it was found that the combination of *NQO1* 3′‐UTR and SERPINA1 mRNA coding region could encode the anti‐trypsin inhibitor‐1 or change the pyridine nucleotide oxidation‐reduction equilibrium (Siegel & Ross, 2000). At the same time, *NQO1* could reduce quinones and their derivatives to hydroquinones by double electron oxidation‐reduction reaction, so that decreased the carcinogenic and teratogenic effects of the compounds, thereby protecting the cells from oxidation to reduction cycles and oxidative stress damage (Long & Jaiswal, 2000; Ross et al., 2000; Sorensen et al., 2003). The above studies showed that high expression of *NQO1* mRNA was involved in the pathophysiological process of METH dependence oxidative stress. Long‐term abuse of METH made the body in a state of oxidative stress for a long time. Maintaining the balance between the internal and external environment should consume more antioxidant enzymes such as *NQO1*. Therefore, the plasma protein level of *NQO1* in METH dependence was significantly lower than controls, which suggested that *NQO1* protein was involved in the mechanism of METH dependence. However, Sun et al. (2018) reported that alcohol treatment could increase *NQO1* protein expression in hepatoblastoma cells, long‐term ethanol treatment increased *NQO1* activity in mice, the results also showed that ethanol‐induced oxidative stress activated the expression of *NQO1* in hepatocytes (Renon et al., 2016; Yeligar et al., 2010). Yun et al. (2015) found that the expression of *NQO1* was increased in the frontal cortex, striatum, and liver of morphine‐dependent mice. We were unable to fully explain this discrepancy. It might be dissimilar protein expression of the *NQO1* contribution to different diseases or tissues. However, the mRNA levels of *NQO1* in PBMCs were not consistent with the plasma protein in METH dependence. This might be associated with the degradation of transcription products or posttranslation modification affects the translation rate of proteins (Belle et al., 2006; Beyer et al., 2004). In yeast and bacteria, 30%‐85% of protein levels can be attributed to changes in mRNA expression, the other 15%‐70% variation is explained by posttranscription or posttranslation regulation and measurement errors, only 40% of proteins in multicellular organisms can be explained by mRNA (de Sousa Abreu et al., 2009). Studies have shown that more than 70% of the protein expression is regulated by mRNA, in yeast, while only about half of the protein expression in *E*. *coli* is regulated by mRNA (Liu et al., 2013; Lu et al., 2007). Besides, we substituted the *NQO1* mRNA levels in PBMCs and plasma protein levels for those of brain tissue, which might be possibly different from that in the brain. Therefore, our findings need to be confirmed in further study, in which the *NQO1* mRNA and plasma protein levels in the brain would be carried out in METH dependence.

In controls, we found that the frequencies of the allele T of *NQO1* 609C/T polymorphism were 41.78%. The allele frequencies in Asian, European, and African American populations were 51.16%, 21.67%, and 17.39%, respectively (https://www.ncbi.nlm.nih.gov/projects/SNP/snp_ref.cgi?do_not_redirect&rs=rs1800566). Our results were not approximate consistent with those of the European population (20.3% in Spanish, Agundez et al., 2014). It might be geographic variations, ethnic, and genetic heterogeneity that could change the frequency of specific polymorphisms in different regions. Therefore, our findings need to be confirmed by a large‐scale study. In this study, it was found that the T allele of *NQO1* 609C/T polymorphism was a risk factor for METH dependence genetic susceptibility, similar results were observed after adjusting for age and gender, which suggested that individuals carrying T allele could increase the risk of METH dependence, and this polymorphism could be used to identify high‐risk individuals. The *NQO1* 609C/T polymorphism was mutated from cytosine to thymine and changed the secondary structure and activity of enzymes, which may impact its biochemical and cellular functions. Mutant protein was rapidly degraded by proteolytic enzyme pathway, and the half‐life was reduced from 18 h to 2 h (Siegel et al., 2001). The 609C/T polymorphism CC genotype has complete enzyme activity, CT genotype enzyme activity decreased and TT genotype enzyme activity was lowest (Ross et al., 1996; Traver et al., 1997). *NQO1* also has an anti‐inflammatory effect (Chen et al., 2003; Iskander et al., 2006) and its 609C/T polymorphism may weaken it. These may increase the risk of METH dependence and the results need to be confirmed by a large‐scale study.

In order to determine the biological function of the *NQO1* 609C/T polymorphism, we analyzed the relationship between *NQO1* 609C/T polymorphism genotype and mRNA and protein. Unfortunately, in current study, individuals carried any genotypes of *NQO1* 609C/T polymorphism did not significantly affect the mRNA in PBMCs and plasma protein levels of *NQO1* in controls. It might be that the carried T genotype of *NQO1* produced a mutant enzyme affects the activity but does not affect mRNA and plasma protein expression (Chen et al., 2004). However, Tijhuis et al. demonstrated that the *NQO1* 609C/T polymorphism has a great effect on rectal *NQO1* mRNA level and enzyme activity (Tijhuis et al., 2008). It might be that *NQO1* 609C/T polymorphism in different diseases has different functions, which need more experimental results to be confirmed.

We failed to find that any genotype of the *NQO1* 609C/T was related to demographic characteristics, routine blood test, or blood pressure under heterozygote and homozygote genetic models, respectively (*p* > 0.05). These findings demonstrated that the aforementioned factors might not contribute to the pathophysiological process of METH dependence in the 609C/T polymorphism. The *NQO1* polymorphism only influenced the responses to METH dependence due to pharmacogenetic difference, but not demographic characteristics, routine blood test, or blood pressure.

There were some limitations of the present study. First, the case–control study was carried out in a single population and a single region, which might result in selection bias and so the results may not be able to be extrapolated to other populations. Further studies could help to establish the true association between the polymorphism and the METH dependence in multi‐family, multi–center, and large samples. Second, only one common functional polymorphism (609C/T) of *NQO1* was investigated, which might lead to some bias, whereas other polymorphisms of *NQO1* were not considered. There might be other gene polymorphisms that contribute to the METH dependence genetic susceptibility. Third, it might result in statistical deviation because the random samples were selected to evaluate the biological function, instead of allele‐transformed cell lines, which need to be carried out through enzyme activity, substrate levels, cell transfection, and animal models to explain the molecular mechanisms of *NQO1* 609C/T polymorphism. Finally, we did not analyze the interaction between genetic polymorphisms and environmental risk factors because of a retrospective study that lacked reliable and sufficient information on other environmental exposures.

In summary, our study suggests that *NQO1* could play an important role in the pathophysiological process of METH dependence, and the 609C/T polymorphism might be associated with increased susceptibility to the development of METH dependence in a Chinese Han population and not affected by age, sex, BMI, blood pressure, and inflammatory status. *NQO1* 609C/T polymorphism did not affect its transcription and translation levels. Confirmation of the role of this *NQO1* polymorphism in METH dependence requires further studies that include large sample sizes and different populations.

## CONFLICT OF INTEREST

None of the authors has any potential financial conflict of interest related to this manuscript.

## AUTHOR CONTRIBUTIONS

H.L. performed all the work and most of the analysis and contributed to the writing of the manuscript; W.Z., X.D.D., and Y.M. helped with the set‐up of some of the molecular methods and with data analysis; and Y.L. designed and supervised the study and contributed to the writing, reviewing, and editing of the manuscript.

## Data Availability

The data that support the findings of this study are available from the corresponding author upon reasonable request.
